# ONC201/dordaviprone for H3 K27M-mutant tumors: Discovery, mechanisms, therapeutic potential, and future directions

**DOI:** 10.1016/j.gendis.2026.102335

**Published:** 2026-06-24

**Authors:** Julio M. Pimentel, Jun-Ying Zhou, Gen Sheng Wu

**Affiliations:** a Cancer Biology Program, Karmanos Cancer Institute, Wayne State University School of Medicine, Detroit, MI 48201, USA; b Departments of Oncology and Pathology, Karmanos Cancer Institute, Wayne State University School of Medicine, Detroit, MI 48201, USA

**Keywords:** ClpP, DRD2, Glioma, H3 K27M mutant, ONC201, Therapy, TRAIL

## Abstract

ONC201 (dordaviprone) has recently received accelerated approval for the treatment of recurrent H3 K27M–mutant diffuse midline glioma. Originally identified as TRAIL-inducing compound 10 (TIC10), ONC201 is a first-in-class small molecule distinguished by a unique pharmacologic profile. It promotes apoptosis through multiple convergent mechanisms, including upregulation of TRAIL, activation of the integrated stress response (ISR), direct allosteric activation of the mitochondrial protease ClpP, and antagonism of dopamine receptor D2 (DRD2). These pathways converge to engage both extrinsic and intrinsic apoptotic programs, disrupt mitochondrial bioenergetics, and reprogram survival signaling, distinguishing ONC201 from conventional cytotoxic and targeted agents. Preclinical studies have demonstrated broad antitumor efficacy across diverse cancer models. Clinically, ONC201 has progressed rapidly through early-phase trials, establishing a favorable safety and pharmacokinetic profile, with sustained biological activity and weekly oral dosing. Pivotal trials in H3 K27M-mutant diffuse midline glioma demonstrated meaningful clinical benefit, leading to accelerated regulatory approval and making it the first therapy to receive such approval for this devastating disease. Ongoing studies are expanding ONC201's clinical applications to hematologic malignancies and gynecologic cancers, as well as to combination regimens. Despite these advances, emerging resistance mechanisms, including ClpP mutations and adaptive activation of the EGFR and MAPK/AKT pathways, highlight the need for biomarker development and rational combination strategies. Together, these insights position ONC201 as both a therapeutic milestone and a platform for future translational oncology innovation.

## Introduction

Cancer remains one of the leading causes of death worldwide, highlighting the critical need for therapeutic strategies that selectively target tumor cells without harming normal cells. Standard chemotherapy/radiotherapy and targeted therapies often face limitations in terms of toxicity, resistance, and limited applicability to specific molecular subtypes.^1^ Despite this, apoptosis represents a therapeutic strategy in which immune-derived death ligands, such as TNF-related apoptosis-inducing ligand (TRAIL), selectively induce apoptotic pathways in cancer cells without harming normal cells.^1^ Therefore, there is an urgent need to develop more effective therapeutic agents that induce apoptosis in cancer while minimizing toxicity to normal cells.

ONC201, a small molecule originally designated TRAIL-inducing compound 10 (TIC10) and later named dordaviprone (Modeyso™), was initially identified in an unbiased cell-based screen for compounds capable of inducing TRAIL.^2^, ^3^, ^4^ ONC201 rapidly distinguished itself from other hits through its unique pharmacologic profile. It triggers apoptosis in a p53-independent manner, lacks genotoxicity, crosses the blood–brain barrier, and demonstrates potent antitumor activity after a single oral dose in preclinical models.^2^, ^3^, ^4^, ^5^ These features, combined with its favorable safety profile, established ONC201 as a first-in-class therapeutic candidate for further investigation.

Since its discovery, ONC201 has become the prototypical member of the imipridone class, with significant advances in understanding its mechanisms of action. Initially recognized for its ability to upregulate TRAIL through protein kinase B (AKT) and extracellular signal-regulated kinase (ERK) inhibition and Forkhead box O3a (FoxO3a) transcription factor activation,^2^^,^^6^ ONC201 has since been shown to impact additional apoptotic pathways, including activation of the integrated stress response (ISR),^7^, ^8^, ^9^ selective antagonism of the dopamine receptor D2 (DRD2)^10^^,^^11^ and direct perturbation of mitochondrial homeostasis via caseinolytic peptidase *p* (ClpP) activation.^12^, ^13^, ^14^ These intersecting pathways enable ONC201 to induce both extrinsic and intrinsic apoptosis, disrupt mitochondrial bioenergetics, and reprogram pro-survival signaling networks, thereby revealing a multifaceted mechanism distinct from that of most conventional therapies.

Building on this mechanistic foundation, extensive preclinical studies have demonstrated ONC201's broad antitumor efficacy across diverse cancer models. ONC201 upregulates TRAIL, induces apoptosis across a range of tumors, restores TRAIL sensitivity in resistant cells, and enhances the effectiveness of standard therapies by suppressing survival pathways. Importantly, its activity on TRAIL signaling, mitochondrial function, ClpP, and DRD2 allows it to overcome multiple mechanisms of therapeutic resistance, further underscoring its translational potential. These preclinical findings have catalyzed rapid clinical development. ONC201 has progressed through multiple early-phase trials, establishing a favorable pharmacokinetic and safety profile and supporting sustained biological activity with weekly oral dosing. This clinical progress has been most notable in patients with H3 K27M-mutant diffuse midline glioma, where recent pivotal trials have shown meaningful clinical benefit, leading to accelerated regulatory approval and establishing ONC201 as the first approved therapy for this fatal disease, which often affects pediatric and adolescent patients. This clinical success highlights the importance of the unique molecular features of H3 K27M-mutant tumors that shape their biology and therapeutic response.

H3 K27M mutations arise from lysine-to-methionine substitutions at position 27 in histone H3 variants (H3.1 or H3.3), resulting in a global reduction of H3K27 trimethylation (H3K27me3) through inhibition of the polycomb repressive complex 2 (PRC2).^15^^,^^16^ Mechanistically, the mutant histone disrupts PRC2-mediated gene silencing, resulting in widespread epigenetic reprogramming and aberrant activation of oncogenic transcriptional programs that drive tumorigenesis and maintain a stem-like, undifferentiated state characteristic of diffuse midline gliomas.^15^^,^^16^ Beyond transcriptional dysregulation, H3 K27M-mutant tumors exhibit altered metabolic and mitochondrial states, as well as increased reliance on stress–response pathways, including the integrated stress response (ISR), which together create targetable vulnerabilities and will be explored in greater mechanistic detail in subsequent sections.^15^^,^^17^ These dependencies are particularly relevant to ONC201, which activates the mitochondrial protease ClpP and induces ISR signaling, thereby exploiting vulnerabilities created by H3 K27M–driven epigenetic reprogramming.^7^, ^8^, ^9^^,^^12^, ^13^, ^14^

Ongoing clinical studies continue to expand its use across solid tumors and combination regimens, reflecting its versatility as both a monotherapy and a synergistic agent. Furthermore, ONC201 has driven the development of a broader imipridone family designed to enhance potency and overcome resistance. Next-generation analogues such as ONC206, ONC212, and ONC213 strengthen integrated stress response (ISR) activation, suppress ERK/AKT signaling, promote ClpP activity, antagonize DRD2 activity, and activate metabolic apoptotic mechanisms, while fluorinated TBP compounds and ClpP-targeting TR analogues further amplify mitochondrial protein degradation and apoptosis. Together, these agents significantly expand the therapeutic landscape beyond ONC201.

This review provides a comprehensive overview of ONC201 development, focusing on four key areas: (i) its mechanisms of action; (ii) preclinical evidence supporting its antitumor activity and capacity to overcome therapeutic resistance; (iii) clinical development, including pharmacokinetics, safety, and emerging indications; and (iv) next-generation ONC201 analogues and structurally related compounds that expand the imipridone therapeutic landscape. Together, these advances highlight ONC201's evolution from a screening hit to a first-in-class therapeutic with broad oncologic potential.

## TRAIL induction

**Figure 1:**
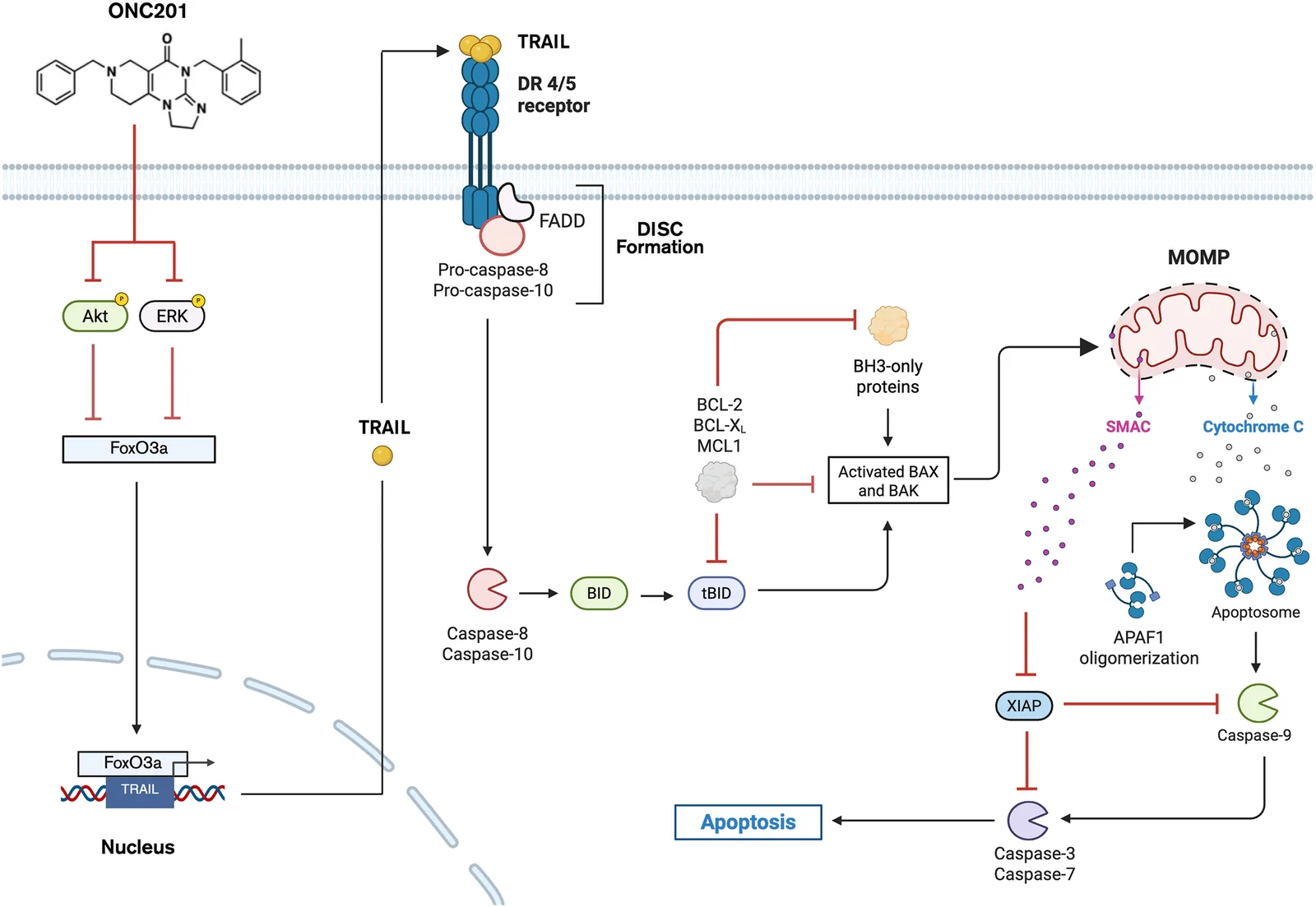
Classical TRAIL signaling induced by ONC201. ONC201 inhibits AKT and ERK signaling, preventing FoxO3a phosphorylation and enabling FoxO3a nuclear translocation. In the nucleus, FOXO3a functions as a transcription factor to induce TRAIL expression. Secreted TRAIL forms a homotrimer that binds death receptor 4 (DR4) or death receptor 5 (DR5), promoting receptor trimerization and activation. TRAIL–DR engagement recruits FADD and procaspase-8/10 to assemble the death-inducing signaling complex (DISC), resulting in caspase-8 activation. Activated caspase-8 cleaves BID to generate tBID, which promotes mitochondrial outer membrane permeabilization (MOMP) through activation of BAX/BAK. This mitochondrial apoptotic step can be suppressed by anti-apoptotic BCL-2 family proteins but is enhanced by BH3-only proteins. MOMP triggers the release of cytochrome c and SMAC/DIABLO, leading to apoptosome formation containing cytochrome c, Apaf-1, and caspase-9, which results in downstream activation of executioner caspases-3/7, culminating in apoptotic cell death. Created with BioRender.com.

Apoptosis preserves tissue homeostasis by eliminating aberrant cells, and its dysregulation is a hallmark of cancer.^1^ Restoring this program is a central therapeutic objective, and one actionable strategy is to re-engage it transcriptionally by elevating endogenous TRAIL, which activates apoptosis via death receptor 4/5 (DR4/DR5), death-inducing signaling complex (DISC) formation, and initiation of a caspase cascade that culminates in cell death^1^ (Fig. 1). Because TRAIL selectively kills cancer cells without harming normal cells,^18^^,^^19^ early efforts focused on recombinant TRAIL and agonist antibodies against DR4/DR5 as TRAIL-based cancer therapies.^20^^,^^21^ These efforts with recombinant TRAIL and agonist antibodies in clinical trials showed that these agents demonstrated limited efficacy in patients with cancer.^22^, ^23^, ^24^ The limited efficacy can be attributed in part to a short serum half-life, variable target engagement, and decoy-receptor interference, while many classical pro-apoptotic drugs rely on genotoxic stress or p53 dependence, limiting selectivity.^1^ Importantly, the lack of efficacy of TRAIL or its agonist antibodies against DR4/5 may also be attributable to the selection of the right patient populations in clinical trials, because only some, but not all, cancer cells are sensitive to the TRAIL pathway.^25^ Therefore, an alternative approach is needed to develop effective TRAIL-based cancer therapeutics, including small molecules that can promote TRAIL-mediated cancer cell death.

Pioneering work by our group addressed this need by showing that diverse anticancer agents can increase TRAIL transcription and trigger DR4/DR5-dependent apoptosis in cancer cells, which established a proof-of-concept strategy and a quantifiable readout for compound discovery.^26^, ^27^, ^28^ These studies laid the foundation for a cell-based screening strategy centered on TRAIL transcriptional output. A further study confirmed that TRAIL induction is a viable approach for killing cancer cells.^29^ Building on this foundation, our TRAIL promoter-driven reporter system was used to screen chemically diverse libraries for compounds that induce tumor-selective apoptosis in a p53-independent manner.^2^^,^^3^

This screening approach ultimately yielded TRAIL-inducing compound 10 (TIC10)—later renamed ONC201—a small molecule that restored apoptotic signaling without genotoxic stress.^2^^,^^3^ ONC201 exhibited a unique pharmacologic profile: it triggered apoptosis independently of p53, crossed the blood–brain barrier, and produced potent antitumor activity following a single oral dose in preclinical models.^2^, ^3^, ^4^ Its onset of activity was typically observed within 36–48 h of treatment, consistent with ONC201 functioning as an effector signaling compound.^3^ By contrast, brefeldin A, an endoplasmic reticulum (ER) stress-inducing compound, also upregulated TRAIL and induced apoptosis but was ultimately excluded as a therapeutic candidate due to its unacceptable toxicity toward normal cells.^3^ Thus, these findings established ONC201 as the most promising candidate from the screen for further development as a cancer therapy.

Initially, it was revealed that ONC201 upregulated TRAIL by inhibiting the phosphorylation of AKT and ERK, leading to reduced phosphorylation of the transcription factor FoxO3a^2^, ^3^, ^4^^,^^6^ (Fig. 1). In its dephosphorylated state, FoxO3a translocates into the nucleus, where it binds directly to the TRAIL promoter and drives p53-independent TRAIL transcription.^2^^,^^6^^,^^30^ This mechanism was functionally validated through RNA interference experiments, which showed that FoxO3a silencing markedly reduced ONC201-induced TRAIL expression and apoptosis, establishing FoxO3a as a key mediator of ONC201's pro-apoptotic activity.^2^ Once secreted, TRAIL assembles into homotrimers that interact with DR4/5 on the tumor cell surface, triggering receptor clustering and DISC formation by recruiting the Fas-associated death domain (FADD) adaptor protein and procaspase-8.^1^^,^^31^ Within the DISC, procaspase-8 undergoes autocatalytic activation to form caspase-8, which culminates in the activation of executioner caspases-3 and 7, resulting in apoptosis and, in preclinical models, tumor regression across several ONC201-treated cancer cell lines.^14^^,^^32^, ^33^, ^34^, ^35^, ^36^, ^37^, ^38^, ^39^

While ONC201's ability to promote TRAIL expression through AKT and ERK inhibition is well established, the extent to which these pathways contribute to additional apoptotic mechanisms remains an active area of investigation. Evidence suggests that ONC201-mediated AKT dephosphorylation can enhance cell death by activating glycogen synthase kinase 3β (GSK3β), thereby suppressing c-Myc and revealing an additional pathway by which ONC201 affects cell viability under diminished AKT signaling.^40^ Collectively, these findings indicate that ONC201's pro-apoptotic effects extend beyond FoxO3a activation to encompass a broader network of downstream effectors within the AKT and ERK signaling axes that remain to be fully elucidated.

## ERK- and AKT-independent TRAIL induction

**Figure 2:**
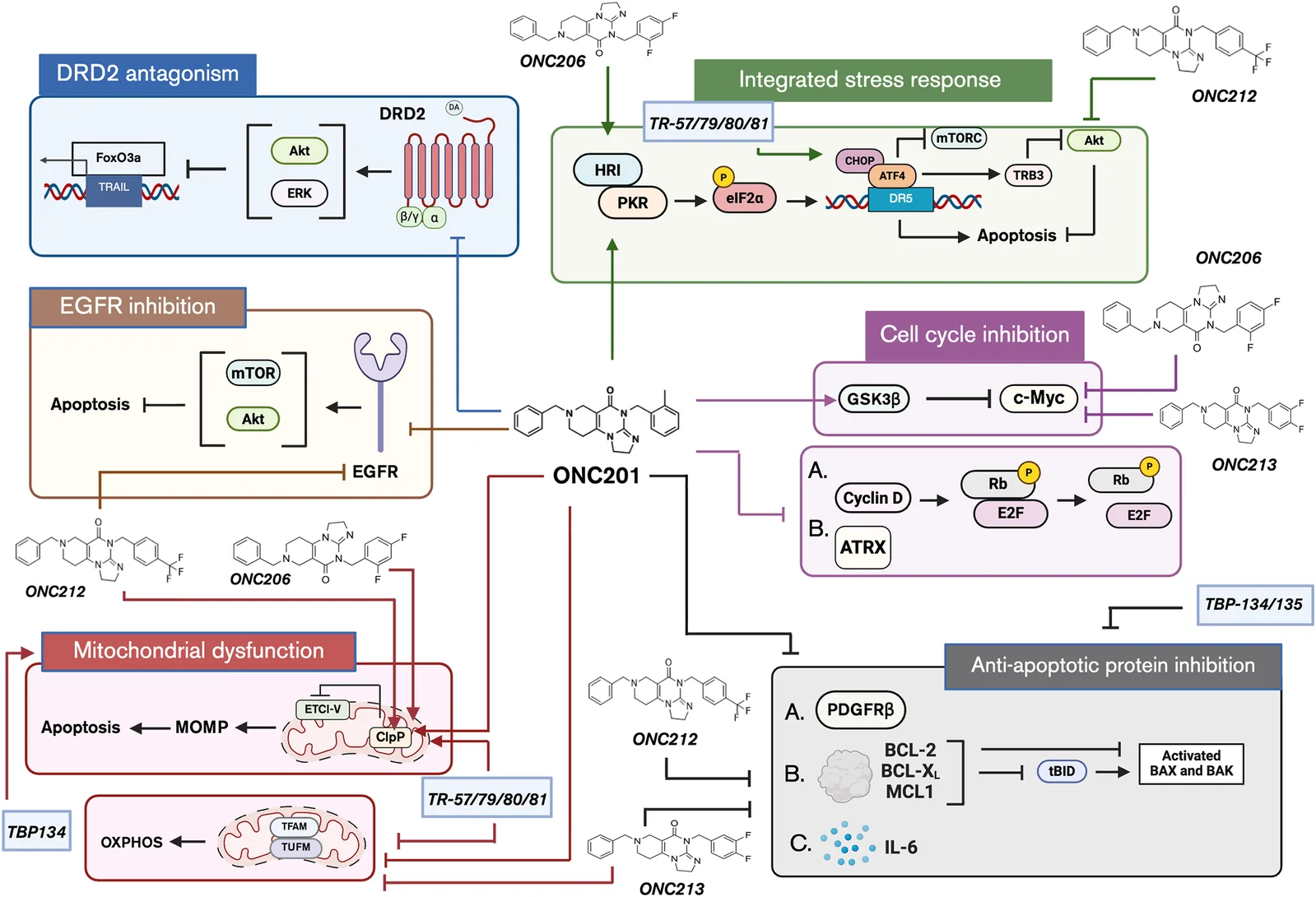
Signaling pathways targeted by ONC201 and next-generation imipridones. ONC201, the original imipridone, induces apoptosis through coordinated effects across multiple cellular pathways. It antagonizes DRD2 to suppress AKT/ERK signaling and promote TRAIL expression via FoxO3a upregulation, activates the integrated stress response (HRI/PKR–eIF2α–ATF4/CHOP) to drive DR5 and inhibit AKT/mTOR signaling through TRB3 and ATF4/CHOP, and disrupts mitochondrial function via ClpP activation and TFAM/TUFM suppression. ONC201 also inhibits EGFR signaling, further suppressing AKT/ERK activity, and disrupts cell cycle progression (GSK3β–c-MYC, cyclin D–RB–E2F, ATRX), while downregulating anti-apoptotic and pro-survival proteins (BCL-2 family proteins, PDGFRβ, IL-6). These mechanisms are conserved and enhanced in next-generation imipridones, including ONC206, ONC212, ONC213, TR compounds (TR-57/79/80/81), and TBP compounds (TBP-134/135), which similarly target stress-response, cell cycle, anti-apoptotic, and mitochondrial pathways to promote apoptotic cell death. Created with BioRender.com.

In addition to the TRAIL/FoxO3a axis, accumulating evidence suggests that ONC201 activates additional apoptotic pathways independent of AKT and ERK signaling^7^, ^8^, ^9^^,^^36^^,^^38^^,^^41^^,^^42^ (Fig. 2). One such mechanism involves the rapid activation of the integrated stress response (ISR) pathway (Fig. 2). In this pathway, the upstream kinases heme-regulated inhibitor (HRI) and protein kinase R (PKR) phosphorylate eukaryotic initiation factor 2α (eIF2α), thereby triggering the induction of activating transcription factor 4 (ATF4) and C/EBP homologous protein (CHOP).^7^ This process occurs in an ERK-independent manner, relying on HRI/PKR signaling in solid tumors and on general control nonderepressible 2 (GCN2) in hematologic malignancies.^7^ Once activated, ATF4 and CHOP orchestrate transcriptional programs that promote apoptosis, most prominently through CHOP-driven upregulation of DR5, which provides a parallel mechanism for engaging the extrinsic apoptotic pathway.^7^^,^^41^^,^^43^^,^^44^

Beyond its role in DR5 regulation, ATF4 signaling has broader effects on cell survival. For example, it has been shown to inhibit mechanistic target of rapamycin complex (mTORC) activity, thereby suppressing downstream pro-survival signaling.^9^ Additionally, ISR signaling upregulates Tribbles homolog 3 (TRB3), a negative regulator of AKT, establishing a mechanistic link between ISR activation and suppression of AKT activity.^45^^,^^46^ ONC201 also downregulates the anti-apoptotic Bcl-2 family protein myeloid cell leukemia 1 (Mcl-1), further sensitizing tumor cells to apoptotic death.^8^ Thus, these ISR-mediated events complement TRAIL/FoxO3a signaling and reinforce ONC201's capacity to promote apoptosis through multiple pathways.

## ONC201-induced ClpP activation and mitochondrial dysfunction

Another pathway through which ONC201 promotes cell death involves the direct disruption of mitochondrial homeostasis by activating the serine protease ClpP^12^, ^13^, ^14^^,^^43^^,^^47^ (Fig. 2), a key regulator of mitochondrial protein quality control.^48^, ^49^, ^50^, ^51^ ClpP was first identified as a direct target of ONC201 through chemical proteomics and library screening approaches.^12^, ^13^, ^14^^,^^43^^,^^47^ Under normal physiological conditions, ClpP functions with its ATP-dependent chaperone, ClpX, to degrade misfolded proteins and maintain mitochondrial integrity.^48^, ^49^, ^50^, ^51^ Substrates of this proteolytic system include key proteins such as Era-like 12S mitochondrial rRNA chaperone 1 (ERAL1) and multiple components of the electron transport chain (ETC).^13^^,^^48^, ^49^, ^50^, ^51^ Upon exposure to ONC201, ClpP binds to the compound noncovalently, inducing an allosteric conformational shift that hyperactivates its proteolytic activity.^12^^,^^13^^,^^47^^,^^52^^,^^53^ Acting independently of ClpX, this altered substrate specificity drives uncontrolled degradation of ETC proteins, leading to impaired oxidative phosphorylation (OXPHOS), loss of mitochondrial bioenergetics, and activation of intrinsic apoptosis (also known as the mitochondrial or caspase-independent pathway).^4^^,^^52^^,^^54^^,^^55^

While mitochondrial dysfunction represents a significant arm of ONC201's intrinsic apoptotic activity, its downstream effects also intersect with signaling pathways that can promote extrinsic-like apoptosis through TRAIL-independent mechanisms. Impaired OXPHOS and the consequent loss of mitochondrial bioenergetics are thought to reduce the phosphorylation of ERK, AKT, and mTOR, as well as of receptor tyrosine kinases such as the epidermal growth factor receptor (EGFR) and the platelet-derived growth factor receptor beta (PDGFRβ).^56^ ONC201 additionally downregulates proteins essential for mitochondrial integrity and transcriptional regulation, including mitochondrial transcription factor A (TFAM).^14^ Complementary studies have shown that ClpP activation by ONC201 disrupts mitochondrial function, triggering ISR and leading to eIF2α phosphorylation, activation of the ATF4/CHOP axis, and apoptotic cell death.^7^^,^^8^^,^^14^^,^^40^ Although promoting cell death through mitochondrial mechanisms highlights the broad therapeutic potential of ONC201, whether ClpP expression and mitochondrial bioenergetics alone are sufficient to predict therapeutic response remains unclear and is under ongoing investigation.

## Nuclear mechanisms of ONC201

Beyond its well-characterized effects on the TRAIL pathway and mitochondrial ClpP activation, the full range of cellular pathways modulated by ONC201 remains unclear. Emerging evidence indicates that ONC201 can also modulate nuclear gene expression, including the nuclear translocation of FoxO3a, which activates the transcription of TRAIL and pro-apoptotic genes, such as CHOP, thereby increasing DR5 expression.^3^^,^^5^^,^^33^ ONC201 has also been shown to reduce cyclin D expression and phosphorylated Rb levels, thereby impairing cell cycle progression.^7^ In neuroblastoma cells, ONC201 treatment reactivates alpha-thalassemia/mental retardation X-linked (ATRX) expression. This chromatin remodeler localizes to the nucleus and plays a key role in maintaining genomic stability and transcriptional regulation.^57^ Concurrently, ONC201 reduces MYCN, an oncogenic transcription factor that promotes cell proliferation.^57^ Together, these transcriptional changes reflect ONC201's capacity to reprogram nuclear chromatin dynamics, shifting gene expression toward a more differentiated, less proliferative state. Therefore, continued investigation into the broader cellular pathways regulated by ONC201 will be essential to fully elucidate its mechanism of action and expand its therapeutic potential.

## ONC201 and dopamine D2 receptor interaction

G-protein-coupled receptors (GPCRs) constitute a large family of cell-surface receptors that regulate various physiological processes and represent primary targets for many FDA-approved drugs, particularly when their signaling becomes dysregulated.^58^, ^59^, ^60^ Dopamine receptors, a subfamily of GPCRs, are implicated in cancer progression, as cancer cells can exploit neurotransmitters like dopamine and serotonin to drive tumor initiation and growth.^11^^,^^61^, ^62^, ^63^ Dopamine plasma levels are often elevated in cancer patients^64^ and can accumulate within the brain, contributing to the formation and growth of brain tumors.^61^^,^^65^ The dopamine receptor family has five receptors, among which ONC201 selectively targets the dopamine D2 receptor (DRD2)^10^^,^^66^ (Fig. 2). DRD2 is a GPCR that regulates neurotransmission by adenylate cyclase activity, thereby modulating dopamine-dependent pathways involved in movement, motivation, reward, and endocrine function.^67^ It can also activate AKT and ERK,^68^^,^^69^ thereby suppressing TRAIL expression.^2^^,^^32^^,^^35^ ONC201 preferentially targets DRD2 rather than other GPCRs because it exhibits a unique dual orthosteric–allosteric binding mode at DRD2 that is not conserved at other receptors, enabling selective antagonism without affecting other dopamine receptor subtypes.^11^ This specificity enables ONC201 to achieve potent anti-tumor efficacy with a broad therapeutic index, distinguishing it from traditional antipsychotics that broadly inhibit DRD2 and related GPCRs while lacking comparable anti-cancer activity.^11^^,^^70^ Further studies are needed to elucidate how dopamine receptors modulate TRAIL upregulation and to determine whether these receptors interact with other proteins or whether additional GPCRs contribute to TRAIL induction.

While these sections highlight the diverse mechanisms by which ONC201 exerts antitumor activity, emerging evidence suggests that these pathways do not contribute equally; instead, they exhibit context-dependent dominance across tumor types.^2^, ^3^, ^4^^,^^7^^,^^12^, ^13^, ^14^ In H3 K27M-mutant gliomas, ClpP-mediated mitochondrial dysfunction and ISR activation represent key drivers of therapeutic response, reflecting the metabolic and epigenetic vulnerabilities that sensitize these tumors to mitochondrial stress and ISR signaling.^7^, ^8^, ^9^^,^^12^, ^13^, ^14^ In contrast, DRD2 antagonism and TRAIL induction may play more context-dependent roles across other tumor types, particularly those with high DRD2 expression or intact apoptotic signaling.^2^, ^3^, ^4^^,^^10^^,^^11^

This context-specific hierarchy highlights the importance of tumor lineage and molecular state in shaping the ONC201 response and underscores the need for biomarker-driven patient stratification. Importantly, these differences in mechanistic dependency have direct implications for downstream apoptotic signaling, including TRAIL-mediated cell death, and may influence the emergence of resistance across tumor types. Understanding how these pathways differentially contribute to therapeutic response will be critical for interpreting variability in clinical outcomes and for guiding the rational development of combination strategies and ongoing clinical trials.

## TRAIL-mediated antitumor activity of ONC201: TRAIL resistance mechanisms and ONC201's role in sensitization

The discovery of ONC201 as a small-molecule inducer of apoptosis by upregulating TRAIL has spurred extensive investigation into its therapeutic index and mechanism of action in cancer. Preclinical studies have demonstrated that ONC201 upregulates TRAIL and induces apoptosis across various cancer models, including cancers of the central nervous system,^2^^,^^34^^,^^56^^,^^71^, ^72^, ^73^, ^74^, ^75^, ^76^, ^77^ lung,^35^^,^^78^^,^^79^ ovarian,^80^, ^81^, ^82^, ^83^ breast,^14^^,^^36^^,^^53^^,^^84^, ^85^, ^86^, ^87^, ^88^ prostate,^37^^,^^39^^,^^79^^,^^89^, ^90^, ^91^ colorectal,^32^^,^^37^^,^^92^, ^93^, ^94^ and uterus,^95^^,^^96^ as well as glioblastoma and hematological malignancies.^8^^,^^38^^,^^42^^,^^97^ In colon, triple-negative breast cancer (TNBC), and glioblastoma cell lines, treatment with ONC201 induced TRAIL mRNA expression within 36–48 h through concurrent inactivation of AKT and ERK signaling. Subsequent nuclear translocation of FOXO3a led to direct activation of the TRAIL promoter, resulting in apoptosis.^2^ *In vivo*, ONC201 upregulates TRAIL and drives tumor regression in TNBC,^2^ lung,^35^ pancreatic,^98^ and colon cancer models,^32^ while crossing the murine blood–brain barrier in central nervous system tumor models to improve survival.^4^^,^^5^ Together, these results highlight the robust antitumor activity of ONC201 in both *in vitro* and *in vivo* cancer models.

The most notable effect of ONC201 is its capacity to restore TRAIL sensitivity in cancer cells that have developed resistance.^36^ While TRAIL triggers pro-apoptotic signaling, it can paradoxically activate the ERK and AKT pathways that drive survival and resistance.^99^ ONC201 counteracts this by reprogramming these pathways, restoring apoptotic sensitivity even in models resistant to recombinant TRAIL, including colon, breast, and glioblastoma.^2^ In breast cancer, which encompasses diverse subtypes with varying TRAIL responses, ONC201 shows comparable efficacy against both epithelial-like and mesenchymal-like TRAIL-resistant forms.^36^ Furthermore, ONC201 reverses acquired TRAIL resistance in TNBC cell lines engineered to model this condition by suppressing ERK and AKT signaling.^36^ Converging evidence indicates that ERK and AKT are central mediators of TRAIL resistance, demonstrating that TRAIL can engage non-apoptotic pathways to sustain tumor cell survival.^99^^,^^100^ In TNBC specifically, TRAIL-induced ERK phosphorylation upregulates the immune checkpoint protein programmed death-ligand 1 (PD-L1), thereby suppressing apoptosis and inducing immune evasion.^100^ Supporting this idea, a recent study showed that ONC201 treatment reduced PD-L1 expression in pancreatic cancers, although the underlying mechanisms remain to be fully elucidated.^101^

Extending beyond its intracellular signaling effects, TRAIL also remodels the tumor microenvironment by promoting the production of cytokines, such as interleukin-6 (IL-6), through ERK- and AKT-dependent pathways.^102^ However, it is not yet known whether ONC201 can act on additional signaling pathways that confer TRAIL resistance beyond ERK and AKT, thereby further enhancing the responsiveness of resistant cells and extending its therapeutic reach. Although ONC201 increases TRAIL expression and caspase-8 activation in cancer cell lines,^2^ it can also induce caspase-independent apoptosis in certain breast, endometrial, and glioblastoma models because its pro-apoptotic mechanism is highly cell context-dependent.^14^^,^^34^ When considered alongside the findings above, these insights identify immune-evasion ligands, pro-inflammatory factors, and other pathways as key mediators and unexplored therapeutic targets for ONC201 that may enable the reversal of TRAIL resistance.

## ONC201 restores therapy sensitivity and synergizes with standard treatments

In addition to overcoming TRAIL resistance, ONC201 can re-sensitize tumors to standard treatments or work synergistically with them across multiple cancer types (Table 1).

**Table 1:** ONC201 combination strategies and their mechanistic effects on tumor cell death.

Cancer Type	Partner Therapy	Effect	Ref.
Glioblastoma; medulloblastoma	Paxalisib	AKT inhibition; ISR activation	56,103
–	2-Deoxyglucose	Metabolic stress	56
–	ABT-263; ABT-199	Mcl-1 inhibition	34,83
–	Temozolomide	Restored sensitivity to bevacizumab and radiation	2,72,77
Pancreatic	5-Fluorouracil	Restored drug sensitivity through ERK/AKT suppression	132
–	Gemcitabine	ERK/AKT suppression	101,145
–	TLY012	Suppression of c-FLIP, Bcl-XL, XIAP, cIAP1, and survivin	44
Acute myeloid leukemia	ABT-199	ATF4 induction	8,146
Multiple myeloma	Bortezomib; carfilzomib	ERK suppression and bim upregulation	97
Hematological malignancies	Bortezomib; Dexamethasone; Cytarabine; 5-azacytidine ixazomib	ATF4/CHOP activation; AKT suppression; downregulation of cyclin D1, IAP, and Bcl-2 family members.	38
ER^+^ breast	Everolimus,	Mitochondrial respiration	88
Triple-negative breast	Trametinib	MEK inhibition	86
BRCA1/2-deficient breast	Rucaparib	PARP1 inhibition	107
Ovarian	Ceralasertib; ABT-263	Alters Mcl-1 and BAG3 expression	83,107
–	Paclitaxel	Restored drug sensitivity by suppressing AKT/ERK, STAT, and NF-κB signaling; activation of unfolded protein response and ER stress pathways.	80
Head and neck	Cisplatin	ATF3/4 and CHOP activation	108
Lung	Carboplatin; Etoposide	ATF activation; PARP1 cleavage	109
–	Paclitaxel; Docetaxel	TRAIL-induced apoptosis	109
–	Lurbinectedin	ATF4 and CHOP activation; γ-H2AX, p-Chk1, and Wee1 increased; PARP cleavage	78
Uterine	Paclitaxel	TRAIL, DR5, ATF4, and CHOP increased; Suppresses AKT/ERK	95
Prostate	Enzalutamide; Darolutamide	ISR activation; AR blockade	90
–	EZH1/2 and HDAC inhibitors	Amplified ISR activation; cell death	91

## Glioblastoma

In glioblastoma cell lines with acquired temozolomide resistance, ONC201 treatment reinstated drug responsiveness^2^ and exhibited cytotoxic activity in patient-derived glioblastoma samples resistant to bevacizumab and radiation.^77^ Co-treatment of ONC201 with the Bcl-2 inhibitors ABT-263 or ABT-199 resulted in a marked decrease in anti-apoptotic proteins such as Mcl-1,^34^^,^^83^ whereas co-treatment with 2-deoxyglucose, a glycolysis inhibitor, further enhanced the cytotoxic effects of ONC201.^56^ Additionally, co-treatment with the AKT inhibitor paxalisib produced synergistic effects and induced mitochondrial damage, further enhancing cytotoxicity in glioblastoma cells.^103^ ONC201 treatment also modulated the tumor microenvironment by altering the production of key cytokines, chemokines, and growth factors, including IL-8, vascular endothelial growth factor (VEGF), and macrophage colony-stimulating factor (M-CSF).^77^^,^^104^^,^^105^

## Colorectal and pancreatic cancer

ONC201 also re-sensitized 5-fluorouracil-resistant colorectal cancer stem/progenitor cells (CSCs) by suppressing ERK and AKT signaling and activating FoxO3a.^32^ Interestingly, ONC201 treatment also reduced the expression of multiple cytokines and chemokines.^106^ A similar response was observed in gemcitabine-resistant pancreatic cancer cells, where ONC201 restored drug sensitivity by inhibiting ERK and AKT.^101^ Synergy was also observed when ONC201 was combined with the TRAIL receptor agonist TLY012 in pancreatic cancer cell lines.^44^

## Hematological malignancy cells and breast cancer cells

In AML cells, the combination of ONC201 and the Bcl-2 antagonist ABT-199 synergistically increased apoptosis.^8^ In multiple myeloma cells resistant to proteasome inhibitors such as bortezomib and carfilzomib, ONC201 restored responsiveness by suppressing ERK and upregulating the pro-apoptotic protein Bim.^97^ Consistently, ONC201 can effectively sensitize cells from hematological malignancies to clinically standard chemotherapies, including bortezomib and dexamethasone.^38^ In therapy-resistant ER^+^ breast cancer cells, co-treatment with ONC201 and the mTOR inhibitor everolimus inhibited c-Myc and reduced cell viability.^88^ Furthermore, the combination of ONC201 and the MEK inhibitor trametinib synergistically inhibited the growth of triple-negative breast cancer cells.^86^ Additionally, combining ONC201 with the PARP1 inhibitor rucaparib produced synergistic effects in BRCA1/2-deficient breast cancer models.^107^

## Ovarian cancer

Platinum resistance in ovarian cancer cells is often driven by the AKT/mTOR and ERK/autophagy survival pathways. ONC201 induced cell death regardless of platinum sensitivity by suppressing AKT/ERK, STAT, and NF-κB signaling while simultaneously activating the unfolded protein response and ER stress pathways, thereby resensitizing platinum-resistant cells to paclitaxel.^80^ ONC201 also effectively inhibited the growth of serous ovarian cancer cells both *in vitro* and *in vivo*.^82^ Moreover, AKT signaling can be further suppressed by combining ONC201 with ABT-263 or ceralasertib, which alters the expression of anti-apoptotic proteins such as Mcl-1 and Bcl-2-associated athanogene 3 (BAG3).^83^^,^^107^

## Head and neck and lung cancers

In head and neck squamous cell carcinoma cells, ONC201 potentiated the therapeutic effect of cisplatin by inducing the ISR transcription factors ATF3, ATF4, and CHOP.^108^ Similar findings were reported in small-cell lung cancer,^109^ where ONC201 co-treatment improved the efficacy of carboplatin and etoposide, accompanied by increased ATF4 expression and cleavage of poly(ADP-ribose) polymerase 1 (PARP1). In non-small cell lung cancer, combining ONC201 with taxanes such as paclitaxel or docetaxel further increased cell death through TRAIL-induced apoptosis.^109^ ONC201 also demonstrated synergistic activity with lurbinectedin in small-cell lung cancer models.^78^

## Uterine and prostate cancer

In uterine carcinoma, a malignancy warranting further exploration with ONC201, ONC201 enhanced the anticancer efficacy of paclitaxel in uterine serous carcinoma cells.^95^ In prostate cancer, combining ONC201 with enzalutamide or darolutamide increased antitumor activity in castration-resistant models by co-targeting the androgen receptor and ISR signaling pathways.^90^ ONC201 also exhibited synergy with everolimus, resulting in increased prostate cancer cell death.^39^ Furthermore, co-treatment with EZH1/2 or HDAC inhibitors amplified ONC201-induced ISR activation and cell death.^91^ While these findings are promising, further studies are needed to fully define ONC201's therapeutic potential. Collectively, these findings indicate that ONC201 has the potential to sensitize therapy-resistant cancers and act synergistically with many drugs.

## Pharmacokinetics and phase I clinical development

The clinical development of ONC201 has expanded considerably in recent years, with multiple trials now evaluating its therapeutic potential in various cancers (Table 2). First-in-human clinical trials established the safety, dosing, and early activity of ONC201.^110^ Subsequently, a number of clinical trials have been launched to evaluate ONC201's anticancer efficacy and safety in cancer patients.^111^, ^112^, ^113^, ^114^ In one phase I study (NCT02250781), weekly oral administration of ONC201 for 3 weeks at doses up to 625 mg was well tolerated and produced robust immunostimulatory effects, including ISR activation and natural killer cell infiltration, resulting in prolonged stable disease in patients with advanced solid tumors, such as glioblastoma, as well as prostate, colon, and endometrial cancer.^105^

**Table 2:** Completed, ongoing, and actively recruiting clinical trials of ONC201.

Trial identifier	Phase	Indication	Status	Comments	Ref.
NCT02250781	I	Advanced solid tumors	Completed	ISR activation; natural killer cell infiltration	105
NCT02609230	I	Solid tumors; myeloma	Completed	Favorable pharmacokinetics; RP2D 625 mg	–
NCT03416530	I	Diffuse intrinsic pontine glioma; H3 K27M-mutant gliomas.	Terminated	Prolonged survival	111,113,114,124,126
NCT03932643	I	AML; MDS	Completed	Safety monitoring	147
NCT05542407	I	Endometrial cancer	Recruiting	Combination of ONC201 and atezolizumab; obesity-driven	–
NCT03932643	I	AML; MDS	Completed	Safety monitoring	–
NCT05630794	I	Colorectal cancer	Recruiting	Testing safety and cancer-preventive effects	–
NCT02324621	I	Advanced Solid tumors	Completed	Well-tolerated and results in enhanced immunostimulatory activity	105
NCT02525692	II	Glioblastoma; H3 K27M mutant diffuse glioma; diffuse midline glioma	Terminated	Limited overall efficacy; ISR (ATF4/CHOP) and TRAIL pathway activation	117,119,124,148
NCT03034200	II	Neuroendocrine (metastatic pheochromocytoma-paraganglioma; desmoplastic small round cell tumors)	Completed	Partial response; durable disease stabilization; DRD2 antagonism; ClpP and ISR activation.	121
NCT03394027	II	Recurrent and metastatic breast cancer; Endometrial cancer	Completed	Stable disease; low adverse effects	122
NCT04617002	II	H3K27M diffuse midline glioma	Approved for marketing	∼30% ORR; durable response; low adverse events; associated with reduced corticosteroid use	124,149
NCT03134131	II	H3 K27M-mutant diffuse glioma; midline high grade glioma	No longer available	∼20% ORR	112,124,126,150
NCT05009992	II	Diffuse intrinsic pontine glioma; diffuse midline glioma; H3 K27M-mutant gliomas	Recruiting	Testing ONC201; radiation; paxalisib	–
NCT02392572	II	Relapsed/refractory AML; high-risk MDS	Recruiting	ONC201; Venetoclax	–
NCT04055649	II	Ovarian cancer	Recruiting	Testing ONC201; paclitaxel	–
NCT03791398	II	Microsatellite Stable (MSS) metastatic colorectal cancer	Terminated	ONC201 plus nivolumab. Efficacy was not observed	123
NCT03733119	II		Terminated	A methionine-restricted diet enhanced ONC201 activity; AKT/ERK signaling was suppressed.	–
NCT05476939	III	Diffuse intrinsic pontine glioma; diffuse midline glioma H3 K27M mutant glioma	Recruiting	ONC201; everolimus; radiation	–
NCT05580562	III	H3 K27M diffuse midline glioma after radiotherapy	Recruiting	Testing clinical efficacy	125

Another phase I trial (NCT02609230) compared weekly versus once-every-three-week oral dosing in patients with advanced solid tumors and multiple myeloma to determine the maximum tolerated dose (MTD) and the recommended phase II dose (RP2D). However, no formal efficacy results have been reported. Overall, the pharmacokinetic and pharmacodynamic profile of ONC201 is favorable, with average *C*_max_ values at tested doses ranging from approximately 1.5 to 7.5 μg/mL, a half-life of about 11.3 h, and an AUC of roughly 37.7 h·μg/mL at the 625 mg dose. These phase I data show that ONC201 is rapidly absorbed, widely distributed in tissues, and cleared within ∼1 day, while its prolonged biological activity supports intermittent oral dosing.^110^

## Phase II clinical evaluation across tumor types

Building on encouraging phase I results and preclinical efficacy, several phase II clinical trials have evaluated ONC201 across diverse tumor types. In one study, ONC201 elicited radiographic responses in patients with recurrent glioblastoma, including those with H3 K27M-mutant diffuse midline glioma (NCT02525692),^115^^,^^116^ establishing the rationale for subsequent clinical trials specifically targeting adult patients with recurrent glioblastoma harboring H3 K27M mutations.^11^^,^^117^, ^118^, ^119^, ^120^ ONC201 has also been evaluated in combination with paxalisib for the treatment of H3K27M-altered diffuse midline glioma.^103^ The combination led to the suppression of AKT and the activation of ClpP, forming the basis for the subsequent clinical trial (NCT05009992).

Beyond glioma, a separate phase II study (NCT03034200) evaluated ONC201 at an oral dose of 625 mg, either once weekly or on two consecutive days per week, in patients with neuroendocrine tumors. Both regimens were well tolerated and achieved clinical benefit, including partial responses and durable disease stabilization in metastatic pheochromocytoma–paraganglioma (PC–PG) and desmoplastic small round cell tumors (DSRCTs).^121^ The dosing schedule that advanced to further trials was 625 mg orally once weekly, as it demonstrated consistent efficacy with a favorable safety profile. Additionally, activation of ISR and ClpP in conjunction with DRD2 antagonism was observed. Another phase II trial (NCT03394027) investigated ONC201 in recurrent or refractory metastatic breast cancer, including HER2-positive and triple-negative subtypes, as well as in advanced endometrial carcinoma.^122^ Although no objective tumor regressions were reported, the study documented a modest 27% clinical benefit rate, largely attributable to stable disease. Notably, the study reaffirmed ONC201's favorable safety profile, which was characterized primarily by low-grade adverse events such as nausea, fatigue, and diarrhea. The recommended phase II dose was again confirmed as 625 mg orally once weekly, consistent with earlier findings. A phase Ib/II clinical trial (Brown University Oncology Research Group; BrUOG 379 [NCT03791398]) was also conducted in microsatellite-stable (MSS) metastatic colorectal cancer (mCRC) patients with a combination of nivolumab plus ONC201; however, efficacy was not observed.^123^ While antitumor activity in these settings was limited, the study provided critical validation of tolerability, refined the dosing strategy, and underscored the need to identify tumor contexts most likely to benefit from ONC201.

## Pivotal trials and regulatory approval in H3 K27M mutant glioma

A more recent pivotal phase II trial in H3 K27M-mutant diffuse midline glioma (NCT04617002) demonstrated a clear clinical benefit. It ultimately provided the basis for accelerated regulatory approval of ONC201, marking its transition from an investigational agent to one granted accelerated approval based on clinical trial evidence.^124^^,^^125^ An integrated analysis of 50 pediatric and adult patients with recurrent H3 K27M mutant diffuse midline glioma treated across four clinical trials and one expanded-access program, reported an objective response rate (ORR) of approximately 20%, which rose to about 30% when responses were assessed using combined Response Assessment in Neuro-Oncology (RANO) criteria for high- and low-grade gliomas (HGG/LGG). Responses were durable, with a median time to response of 8.3 months and a median duration of 11.2 months, providing meaningful benefit in a disease that is otherwise uniformly fatal.^125^ ONC201 was also well tolerated, with predominantly low-grade adverse events, and was associated with reductions in corticosteroid use and maintenance or improvement of performance status.

Another critical study showed that ONC201 has clinical efficacy in patients with H3K27M-mutant diffuse midline glioma, with the underlying mechanism being that ONC201 disrupts integrated metabolic and epigenetic pathways and reverses the pathognomonic reduction of H3K27me3.^126^ This study draws from two clinical trials (NCT03416530 and NCT03134131). In addition, another clinical study showed ONC201's efficacy in 174 patients with thalamic tumors.^127^ Together with evidence of target engagement and biological activity, these compelling findings provide the clinical foundation for ONC201's accelerated approval, establishing its role as the first therapy to receive accelerated approval for recurrent H3 K27M-mutant diffuse midline glioma.

## ONC201 analogues: ONC206, ONC212, ONC213

ONC201 is the founding member and first-in-class small molecule of the imipridone family that combines DRD2 antagonism with the activation of ISR, TRAIL/DR5 induction, and mitochondrial ClpP activation, but its modest potency and limited exposure in some settings motivated analogue development. ONC206 (a difluorobenzyl imipridone) and ONC212 (a trifluoromethylbenzyl imipridone) emerged as lead candidates based on their greater nanomolar potency, broader therapeutic windows, and improved pharmacokinetic properties compared with ONC201.^41^^,^^128^ Subsequent mechanistic work demonstrated that these analogues enhanced key ONC201 mechanisms, including DR5/TRAIL induction, suppression of AKT/ERK signaling, mitochondrial dysfunction via ClpP hyperactivation, and DRD2 antagonism.^13^^,^^52^^,^^128^ This resulted in improved efficacy in tumors with high DRD2 expression or OXPHOS dependence,^13^^,^^52^ providing a strong therapeutic rationale for developing ONC206 and ONC212 as next-generation imipridones to overcome primary and acquired ONC201 resistance and broaden clinical applicability.

The increased efficacy of ONC206 and ONC212 is linked to halogen substitutions on the benzyl R1 group of the imipridone scaffold, resulting in greater potency with a wider *in vitro* therapeutic window than ONC201.^13^^,^^52^ ONC206, in particular, has shown robust antitumor effects in uterine serous carcinoma, melanoma, and endometrial cancer models, where it activates ISR signaling, suppresses pro-survival pathways, inhibits migration and invasion *in vitro*, and significantly inhibits tumor growth in xenografts.^40^^,^^129^^,^^130^ Additional work in several cancers, including ovarian cancer, glioblastoma, and medulloblastoma models, confirms that ONC206 triggers mitochondrial dysfunction, ClpP-dependent OXPHOS dysfunction, and tumor cell death, with strong *in vivo* activity in brain tumor models relevant to DMG with an H3K27M mutation.^40^^,^^126^^,^^129^, ^130^, ^131^ GBM cells with high c-Myc expression exhibited increased apoptosis following ONC206 treatment, suggesting c-Myc as a potential predictive biomarker.^40^

ONC212 similarly exhibits a comparable therapeutic window, with studies in colorectal, melanoma, glioblastoma, liver, skin, breast, ovarian, and lymphoma cancer models demonstrating superior efficacy relative to ONC201 and minimal associated adverse effects ^41^^,^^132^, ^133^, ^134^. ONC212 activates ClpP and the ISR pathway, leading to the upregulation of TRAIL and DR5 by inhibiting AKT and ERK signaling.^41^^,^^52^ In pancreatic cancer models, ONC212 acts synergistically with standard chemotherapies, including 5-fluorouracil, irinotecan, oxaliplatin, and the RTK inhibitor crizotinib. It induces cell death in ONC201-resistant cell lines by inhibiting the insulin-like growth factor 1 receptor (IGF1-R).^132^ Furthermore, ONC212 induces apoptosis in AML cells by activating ClpP and stimulating the ISR via GPR132, a GPCR that may serve as a predictive biomarker of ONC212 efficacy.^135^ In addition, high GPR132 expression in pancreatic cancer creates a therapeutic vulnerability that ONC212 exploits, activating GPR132 to drive lethal ISR signaling and promote TRAIL-mediated apoptosis, thereby reducing tumor growth and proliferation in preclinical models.^135^

In parallel, ONC213 has emerged as a mechanistically distinct imipridone analogue that targets mitochondrial metabolism rather than DRD2/TRAIL or R1-driven receptor signaling.^136^ Preclinical studies in AML models have demonstrated that ONC213 directly inhibits α-ketoglutarate dehydrogenase (αKGDH), suppresses OXPHOS, induces mitochondrial stress, reduces Mcl-1 expression, and triggers apoptosis in both bulk tumor cells and leukemia stem cells.^136^^,^^137^ This positions ONC213 as a metabolically targeted imipridone with particular relevance to hematologic malignancies rather than solid tumors.

Whereas ONC201 has advanced into multiple phase I/II trials in primary and recurrent gliomas and other solid tumors, with FDA Fast Track and Orphan Drug designations and approval for glioblastoma, the clinical development of its analogues is at an earlier stage and is currently centered on ONC206. First-in-human phase I studies of oral ONC206 in adults with recurrent and rare primary CNS neoplasms (NCT04541082) and in children and young adults with newly diagnosed or recurrent DMG/high-grade CNS tumors (PNOC023; NCT04732065) are currently enrolling. These trials incorporate dose-escalation and target-validation components designed to define the maximum tolerated dose and pharmacokinetics. As of 2025, ONC212 remains in preclinical and translational development, with strong *in vivo* efficacy and minimal toxicity reported in pancreatic cancer and AML, but no registered clinical trials have yet been listed on *ClinicalTrials*.*gov*. Unlike ONC206 and ONC212, ONC213 has not yet been evaluated in solid tumor or CNS tumor models. Collectively, these studies provide a strong foundation for the clinical evaluation of these analogues, with further investigation warranted across additional cancer types and in combination with other therapeutic modalities.

## Next-generation ONC201 compounds: TBP-134, TBP-135, TR-79, TR-80, and TR-81

Multiple next-generation ONC201 analogues have been developed to address key pharmacologic and mechanistic limitations of ONC201 while complementing the activity of existing analogues. These analogues fall into two major families: (1) fluorinated ONC201 analogues, including the mono-fluorinated TBP-134 and para-fluorinated TBP-135, and (2) ClpP-targeting tracer analogues, such as TR-79, TR-80, and TR-81.^138^ Both fluorinated ONC201 analogues similarly regulate TRAIL and DR signaling pathways as ONC201.^138^ TBP-134 exhibits the highest antiproliferative potency by inducing intrinsic apoptosis, driven by mitochondrial dysfunction and characterized by reduced expression of the anti-apoptotic proteins cellular inhibitor of apoptosis protein 1 (cIAP-1) and X-linked inhibitor of apoptosis protein (XIAP).^138^

In contrast, TBP-135 activates both intrinsic and extrinsic apoptosis, with caspase-3/7 activation and downregulation of XIAP and survivin.^138^ TBP-134 and TBP-135 have been studied in melanoma, as well as in pancreatic, colorectal, lung, and hepatocellular cancer.^138^ Although both analogues show promising pre-clinical activity, they have not yet advanced to clinical testing. The other analogues, TR-series compounds (TR-57, TR-79, TR-80, and TR-81), are amine-containing analogues that bind directly to ClpP, the same mitochondrial protease targeted by ONC201.^12^^,^^87^^,^^139^ In addition, TR-series compounds have been shown to promote the degradation of mitochondrial proteins such as TUFM and TFAM, thereby activating the ATF4/CHOP-mediated integrated stress response.^12^ They also induce growth arrest and ISR activation in breast (TNBC), lung, prostate, and pancreatic cancer models. However, *in vivo* studies have not yet been reported, and no clinical trials evaluating TR-series compounds are currently available.

## Ongoing challenges

Despite significant advances achieved with ONC201, several key challenges and opportunities remain to fully harness its therapeutic potential. A major challenge moving forward is understanding and overcoming both intrinsic and acquired resistance to ONC201, which has emerged as a recurring obstacle across multiple cancer types (Table 3). These resistance mechanisms can be broadly categorized into on-target and bypass mechanisms, and may arise from pre-existing or acquired adaptations (Table 3). On-target resistance results from alterations in the direct molecular targets of ONC201, compromising its therapeutic efficacy. For example, mutations in ClpP impair ONC201-induced proteolytic activation, thereby reducing downstream ISR signaling.^13^ In contrast, bypass resistance involves compensatory activation of alternative survival pathways, including EGFR, MAPK, and AKT signaling, which sustain tumor cell viability independently of ONC201 target engagement.^42^^,^^56^^,^^71^^,^^132^^,^^141^^,^^140^ Furthermore, these resistance mechanisms may be present before treatment, contributing to intrinsic resistance, or emerge during therapy as acquired resistance, reflecting the dynamic and adaptive nature of tumor signaling networks.^56^^,^^141^ Thus, this framework provides a basis for interpreting resistance patterns and for guiding the development of rational combination strategies.

**Table 3:** Resistance mechanisms and counterstrategies.

Mechanism	Biological Basis	Countermeasure	Ref.
EGFR signaling	MAPK/AKT feedback	EGFR inhibitors	142
AKT/MAPK signaling	Adaptive survival	AKT/MEK inhibitors	140
ClpP mutation	Loss of mitochondrial proteolysis; reduced ISR	ONC206/ONC212	144
DRD5 expression	Inhibits DRD2 signaling	DRD5 inhibitor	11
IGF1-R upregulation	Survival signaling	IGF-1R inhibitors (AG1024)	132
EGFR coupling	Survival signaling	EGFR inhibitors	71
NF-κB/JAK-STAT signaling	Increased cFLIP, BCL-2	NF-κB/JAK inhibitors	42
Metabolic adaptation	Restored OXPHOS	2-DG, metabolic inhibitors	56,141

Preclinical studies have demonstrated that cancer cells can adapt to ONC201 exposure by compensating through the activation of pro-survival signaling pathways. Pancreatic cancer models, for example, upregulate insulin-like growth factor 1 receptor (IGF1-R) signaling as a resistance mechanism, a phenomenon that can be reversed by co-treatment with IGF1-R inhibitors such as AG1024.^132^ In H3K27M-mutant diffuse midline glioma, EGFR signaling confers resistance to ONC201,^142^ while single-cell and spatial transcriptomic analyses reveal that diffuse midline glioma cells can acquire resistance through upregulation of MAPK^143^ or AKT signaling.^140^ Additionally, mutations in ClpP that impair imipridone-induced proteolytic activation have been identified in gliomas with acquired resistance, leading to metabolic reprogramming and reduced ISR activation.^144^ In addition, mutations in the dopamine receptor subtype DRD5 can lead to resistance to ONC201.^11^ These findings collectively highlight the adaptive plasticity of tumor signaling pathways and underscore the need for systematic, multi-omic mapping of resistance nodes. Such efforts will be essential for designing rational combination therapies; for example, integrating ONC201 with PI3K, MAPK, DRD5, or EGFR inhibitors to sustain durable responses and prevent therapeutic resistance.

A second major challenge is defining biomarkers of ONC201 sensitivity. ONC201 targets multiple apoptotic and stress pathways—including TRAIL/FoxO3a activation, ISR induction, DRD2 antagonism, and ClpP-mediated mitochondrial dysfunction. However, the relative contribution of these mechanisms varies by tumor lineage, genetic background, and metabolic state. Furthermore, whether parameters such as ClpP expression/activity, mitochondrial bioenergetics, ATF4–CHOP signaling intensity, or DRD2 receptor status can serve as reliable biomarkers remains unresolved. Additionally, it is unclear which of these pathways predominate in determining treatment response or resistance across tumor contexts. Integration of multi-omic profiling (transcriptomic, proteomic, and metabolomic) with clinical response data from ongoing and future trials could enable the development of predictive molecular signatures that guide patient selection and therapeutic optimization. Functional assays that capture pharmacodynamic changes, such as ISR activation, DRD2 occupancy, or TRAIL/DR5 upregulation, should also be incorporated into early-phase studies to refine dosing and enable real-time response monitoring.

A third challenge involves optimizing ONC201 dosing, scheduling, and combination strategies to maximize therapeutic benefit. Although ONC201 demonstrates favorable oral bioavailability and blood–brain barrier penetration, its optimal dosing frequency and integration with standard therapies remain to be defined. Ongoing studies are testing whether intermittent or continuous dosing best sustains ISR activation, and whether ONC201 synergizes most effectively when administered before, concurrently with, or after radiation, chemotherapy, or immune checkpoint blockade. Despite these advances, patient-to-patient variability in pharmacokinetics and pharmacodynamics, absorption, metabolism, or ClpP/DRD2 expression, complicates dose standardization. Thus, systematically addressing these pharmacologic variables through population-based modeling and adaptive trial design will be critical for establishing consistent clinical efficacy.

## Conclusions and future directions

Over the past decade, ONC201 has evolved from a TRAIL-inducing compound identified through unbiased screening into a first-in-class therapeutic with broad antitumor potential. Mechanistically, ONC201 acts through a multifaceted network of pathways, including TRAIL activation, ISR induction, DRD2 antagonism, and ClpP-mediated mitochondrial dysfunction, thereby triggering apoptosis via both extrinsic and intrinsic apoptotic mechanisms. These pleiotropic effects distinguish ONC201 from conventional targeted or cytotoxic agents and provide a strong rationale for its development across diverse cancer types. Preclinical studies have demonstrated its ability to induce apoptosis, restore TRAIL sensitivity, and synergize with standard treatments in a variety of tumor models, while early-phase clinical trials have confirmed its favorable pharmacokinetic and safety profile. Its accelerated regulatory approval for H3 K27M-mutant diffuse midline glioma represents a landmark achievement, marking the first regulatory approval of a therapy for this aggressive disease.

In parallel with ONC201's clinical application, second-generation imipridone analogues such as ONC206, ONC212, and ONC213 have been developed to enhance potency, optimize pharmacologic properties, and potentially overcome primary or acquired resistance to ONC201. ONC206 retains the imipridone scaffold but exhibits greater nanomolar potency, more robust activation of the ISR, and more potent inhibition of pro-survival ERK and AKT signaling while preserving DRD2 antagonism and ClpP-mediated mitochondrial dysfunction. ONC212, by contrast, functions as a dual activator of mitochondrial ClpP and the orphan G-protein-coupled receptor GPR132, producing rapid disruption of OXPHOS, ATF4/CHOP induction, and apoptotic signaling in metabolically vulnerable tumors. In contrast, ONC213 has emerged as a mechanistically distinct imipridone analogue that primarily targets mitochondrial metabolism by inhibiting α-ketoglutarate dehydrogenase and suppressing OXPHOS, thereby inducing mitochondrial stress and apoptosis, particularly in hematologic malignancies. Preclinical studies across multiple solid and hematologic malignancies consistently demonstrate that ONC206 and ONC212 exert broader and deeper antitumor activity than ONC201 *in vitro* and *in vivo*, positioning these agents as complementary members of an emerging imipridone class rather than simple successors to ONC201. Beyond imipridones, fluorinated ONC201 derivatives (TBP-134 and TBP-135) and TR-series ClpP agonists (TR-79, TR-80, TR-81) highlight the therapeutic potential of structurally diverse ONC201-based compounds to induce mitochondrial proteotoxicity, ISR activation, and apoptosis across multiple solid tumors. Thus, these analogues expand ONC201's therapeutic reach and offer additional avenues for targeting tumor vulnerabilities.

Despite these advances, several challenges remain. Resistance mechanisms, including activation of EGFR and MAPK/AKT signaling and mutations in ClpP, underscore the need for a systematic understanding of adaptive signaling networks and for the design of rational combination strategies to sustain and broaden ONC201's efficacy.^140^^,^^142^^,^^143^ In parallel, identifying robust biomarkers that predict ONC201 response will be critical for optimizing patient selection and improving clinical outcomes. Candidate biomarkers such as ClpP abundance and activity, ISR (ATF4–CHOP) and FoxO3a/TRAIL transcriptional signatures, DR4/DR5 expression, and DRD2 receptor status, integrated with pharmacodynamic readouts from on-treatment biopsies, may help refine dosing, timing, and response assessment. However, biomarker development alone is insufficient; refining treatment schedules, addressing tumor heterogeneity and microenvironmental factors, and ensuring long-term safety in combination settings will also be essential to expanding ONC201's clinical utility.

Concurrently, a growing number of clinical trials are evaluating ONC201 across multiple malignancies and therapeutic contexts. These include studies with 1) atezolizumab, a PD-L1 inhibitor, for obesity-driven endometrial cancer (NCT05542407, phase I); 2) adjuvant therapy following radiotherapy in H3 K27M-mutant diffuse glioma (ACTION study; NCT05580562, phase III); 3) H3 K27M diffuse intrinsic pontine glioma and diffuse midline glioma (BIOMEDE 2.0 study; NCT05476939, phase III); 4) relapsed/refractory acute leukemia and high-risk MDS (NCT02392572, phase II); 5) part of a novel combination treatment strategy for diffuse midline gliomas (NCT05009992, phase II); 6) weekly paclitaxel in platinum-refractory or -resistant ovarian cancer (NCT04055649, phase II); 7) evaluating ONC201 under methionine-restriction conditions in triple-negative breast cancer (NCT03733119, phase II); and 8) safety and preventive effects in colorectal cancer and adenomatous polyps (NCT05630794, phase II). Collectively, these trials will define the disease settings and therapeutic combinations in which ONC201 has the most significant clinical impact.

Despite its accelerated regulatory approval for H3 K27M mutant diffuse midline glioma, the full therapeutic scope and mechanistic depth of ONC201 have yet to be realized. Future studies should assess efficacy in additional H3K27-altered tumors, including rare, non-midline H3 K27M contexts, and rigorously evaluate rational combinations with chemotherapy, radiotherapy, and immune checkpoint blockade. Careful optimization of dose, fractionation, and sequencing will be essential to maximize ISR and DR5 activation while minimizing toxicity. Ultimately, integrating mechanistic insights with biomarker-driven trial design and adaptive therapeutic strategies will be pivotal to harnessing ONC201's full potential as a transformative oncology agent capable of reshaping how apoptosis, stress signaling, and immune modulation are therapeutically leveraged.

Looking ahead, several critical directions warrant focused investigation. The full spectrum of signaling pathways modulated by ONC201 remains incompletely defined, particularly in TRAIL-resistant cancers where survival signaling is maintained through death receptor (DR) downregulation, overexpression of anti-apoptotic proteins, or compensatory MAPK/AKT activation. Whether ONC201 directly or indirectly suppresses these mechanisms via upstream regulators remains unclear. Future studies should dissect how ONC201's GPCR-modulatory activity via DRD2 antagonism influences broader signaling networks that integrate apoptosis, metabolic stress, and inflammatory responses. Similarly, its role in modulating tumor immune surveillance, potentially through regulation of PD-L1 expression, cytokine secretion, or antigen presentation, remains largely unexplored. Another emerging frontier involves ONC201's effect on autophagy and cancer stem cell maintenance, both of which could contribute to therapeutic resistance, tumor dormancy, or recurrence. Addressing these gaps through multi-omic, phosphoproteomic, and single-cell approaches, coupled with longitudinal liquid biopsy analyses, will illuminate ONC201-regulated pathways and guide rational therapeutic combinations.

## CRediT authorship contribution statement

**Julio M. Pimentel:** Writing – review & editing, Writing – original draft, Conceptualization. **Jun-Ying Zhou:** Writing – review & editing. **Gen Sheng Wu:** Conceptualization, Funding acquisition, Supervision, Writing – original draft, Writing – review & editing.

## Funding

This work was, in part, supported by P30CA022453 from the NIH.

## Conflict of interests

GS Wu is the co-inventor on the ONC201 patent. GS Wu is a member of *Genes & Diseases* Editorial Board. To minimize bias, GS Wu was excluded from all editorial decision-making related to the acceptance of this article for publication. The remaining authors declare no competing financial interests.

## References

[1] Pimentel J.M., Zhou J.Y., Wu G.S. (2023). The role of TRAIL in apoptosis and immunosurveillance in cancer. Cancers (Basel).

[2] Allen J.E., Krigsfeld G., Mayes P.A. (2013). Dual inactivation of Akt and ERK by TIC10 signals Foxo3a nuclear translocation, TRAIL gene induction, and potent antitumor effects. Sci Transl Med.

[3] Allen J.E., Krigsfeld G., Patel L. (2015). Identification of TRAIL-inducing compounds highlights small molecule ONC201/TIC10 as a unique anti-cancer agent that activates the TRAIL pathway. Mol Cancer.

[4] Allen J.E., Kline C.L.B., Prabhu V.V. (2016). Discovery and clinical introduction of first-in-class imipridone ONC201. Oncotarget.

[5] Allen J.E., Crowder R., El-Deiry W.S. (2015). First-In-class small molecule ONC201 induces DR5 and cell death in tumor but not normal cells to provide a wide therapeutic index as an anti-cancer agent. PLoS One.

[6] Yang J.Y., Zong C.S., Xia W. (2008). ERK promotes tumorigenesis by inhibiting FOXO3a *via* MDM2-mediated degradation. Nat Cell Biol.

[7] Kline C.L., Van den Heuvel A.P., Allen J.E., Prabhu V.V., Dicker D.T., El-Deiry W.S. (2016). ONC201 kills solid tumor cells by triggering an integrated stress response dependent on ATF4 activation by specific eIF2α kinases. Sci Signal.

[8] Ishizawa J., Kojima K., Chachad D. (2016). ATF4 induction through an atypical integrated stress response to ONC201 triggers p53-independent apoptosis in hematological malignancies. Sci Signal.

[9] Endo Greer Y., Lipkowitz S. (2016). ONC201: stressing tumors to death. Sci Signal.

[10] Madhukar N.S., Khade P.K., Huang L. (2019). A Bayesian machine learning approach for drug target identification using diverse data types. Nat Commun.

[11] Prabhu V.V., Madhukar N.S., Gilvary C. (2019). Dopamine receptor D5 is a modulator of tumor response to dopamine receptor D2 antagonism. Clin Cancer Res.

[12] Graves P.R., Aponte-Collazo L.J., Fennell E.M.J. (2019). Mitochondrial protease ClpP is a target for the anticancer compounds ONC201 and related analogues. ACS Chem Biol.

[13] Ishizawa J., Zarabi S.F., Davis R.E. (2019). Mitochondrial ClpP-mediated proteolysis induces selective cancer cell lethality. Cancer Cell.

[14] Greer Y.E., Porat-Shliom N., Nagashima K. (2018). ONC201 kills breast cancer cells*in vitro*by targeting mitochondria. Oncotarget.

[15] Sarthy J.F., Meers M.P., Janssens D.H. (2020). Histone deposition pathways determine the chromatin landscapes of H3.1 and H3.3 K27M oncohistones. Elife.

[16] Harutyunyan A.S., Krug B., Chen H. (2019). H3K27M induces defective chromatin spread of PRC2-mediated repressive H3K27me2/me3 and is essential for glioma tumorigenesis. Nat Commun.

[17] Chung C., Sweha S.R., Pratt D. (2020). Integrated metabolic and epigenomic reprograming by H3K27M mutations in diffuse intrinsic pontine gliomas. Cancer Cell.

[18] Wiley S.R., Schooley K., Smolak P.J. (1995). Identification and characterization of a new member of the TNF family that induces apoptosis. Immunity.

[19] Pitti R.M., Marsters S.A., Ruppert S., Donahue C.J., Moore A., Ashkenazi A. (1996). Induction of apoptosis by apo-2 ligand, a new member of the tumor necrosis factor cytokine family. J Biol Chem.

[20] Ashkenazi A., Pai R.C., Fong S. (1999). Safety and antitumor activity of recombinant soluble Apo2 ligand. J Clin Invest.

[21] Ashkenazi A., Holland P., Eckhardt S.G. (2008). Ligand-based targeting of apoptosis in cancer: the potential of recombinant human apoptosis ligand 2/tumor necrosis factor–related apoptosis-inducing ligand (rhApo2L/TRAIL). J Clin Oncol.

[22] von Karstedt S., Montinaro A., Walczak H. (2017). Exploring the TRAILs less travelled: TRAIL in cancer biology and therapy. Nat Rev Cancer.

[23] Ralff M.D., El-Deiry W.S. (2018). TRAIL pathway targeting therapeutics. Expert Rev Precis Med Drug Dev.

[24] Snajdauf M., Havlova K., Vachtenheim J. (2021). The TRAIL in the treatment of human cancer: an update on clinical trials. Front Mol Biosci.

[25] Rahman M., Davis S.R., Pumphrey J.G. (2009). TRAIL induces apoptosis in triple-negative breast cancer cells with a mesenchymal phenotype. Breast Cancer Res Treat.

[26] Xu J., Zhou J.Y., Wu G.S. (2006). Tumor necrosis factor–related apoptosis-inducing ligand is required for tumor necrosis factor α–mediated sensitization of human breast cancer cells to chemotherapy. Cancer Res.

[27] Xu J., Zhou J.Y., Tainsky M.A., Wu G.S. (2007). Evidence that tumor necrosis factor–related apoptosis-inducing ligand induction by 5-aza-2′-deoxycytidine sensitizes human breast cancer cells to adriamycin. Cancer Res.

[28] Xu J., Zhou J.Y., Wei W.Z., Philipsen S., Wu G.S. (2008). Sp1-mediated TRAIL induction in chemosensitization. Cancer Res.

[29] Kuribayashi K., Krigsfeld G., Wang W. (2008). *TNFSF10* (TRAIL), a p53 target gene that mediates p53-dependent cell death. Cancer Biol Ther.

[30] Modur V., Nagarajan R., Evers B.M., Milbrandt J. (2002). Foxo proteins regulate tumor necrosis Factor-related apoptosis inducing ligand expression implications for pten mutation in prostate cancer. J Biol Chem.

[31] Yuan X., Gajan A., Chu Q., Xiong H., Wu K., Wu G.S. (2018). Developing TRAIL/TRAIL death receptor-based cancer therapies. Cancer Metastasis Rev.

[32] Prabhu V.V., Allen J.E., Dicker D.T., El-Deiry W.S. (2015). Small-molecule ONC201/TIC10 targets chemotherapy-resistant colorectal cancer stem–like cells in an Akt/Foxo3a/TRAIL–dependent manner. Cancer Res.

[33] Talekar M.K., Allen J.E., Dicker D.T., El-Deiry W.S. (2015). ONC201 induces cell death in pediatric non-hodgkin’s lymphoma cells. Cell Cycle.

[34] Karpel-Massler G., Bâ M., Shu C. (2015). TIC10/ONC201 synergizes with Bcl-2/Bcl-xL inhibition in glioblastoma by suppression of Mcl-1 and its binding partners *In vitro* and *in vivo*. Oncotarget.

[35] Feng Y., Zhou J., Li Z., Jiang Y., Zhou Y. (2016). Small molecular TRAIL inducer ONC201 induces death in lung cancer cells: a preclinical study. PLoS One.

[36] Yuan X., Kho D., Xu J., Gajan A., Wu K., Wu G.S. (2017). ONC201 activates ER stress to inhibit the growth of triple-negative breast cancer cells. Oncotarget.

[37] Prabhu V.V., Lulla A.R., Madhukar N.S. (2017). Cancer stem cell-related gene expression as a potential biomarker of response for first-in-class imipridone ONC201 in solid tumors. PLoS One.

[38] Prabhu V.V., Talekar M.K., Lulla A.R. (2018). Single agent and synergistic combinatorial efficacy of first-in-class small molecule imipridone ONC201 in hematological malignancies. Cell Cycle.

[39] Lev A., Lulla A.R., Ross B.C. (2018). ONC201 targets AR and AR-V7 signaling, reduces PSA, and synergizes with everolimus in prostate cancer. Mol Cancer Res.

[40] Ishida C.T., Zhang Y., Bianchetti E. (2018). Metabolic reprogramming by dual AKT/ERK inhibition through imipridones elicits unique vulnerabilities in glioblastoma. Clin Cancer Res.

[41] Wagner J., Kline C.L., Ralff M.D. (2017). Preclinical evaluation of the imipridone family, analogs of clinical stage anti-cancer small molecule ONC201, reveals potent anti-cancer effects of ONC212. Cell Cycle.

[42] Ni X., Zhang X., Hu C.H. (2017). ONC201 selectively induces apoptosis in cutaneous T-cell lymphoma cells *via* activating pro-apoptotic integrated stress response and inactivating JAK/STAT and NF-κB pathways. Oncotarget.

[43] Prabhu V.V., Morrow S., Rahman Kawakibi A. (2020). ONC201 and imipridones: Anti-cancer compounds with clinical efficacy. Neoplasia.

[44] Jhaveri A.V., Zhou L., Ralff M.D. (2021). Combination of ONC201 and TLY012 induces selective, synergistic apoptosis *in vitro* and significantly delays PDAC xenograft growth *in vivo*. Cancer Biol Ther.

[45] Du K., Herzig S., Kulkarni R.N., Montminy M. (2003). TRB3: atribbleshomolog that inhibits Akt/PKB activation by insulin in liver. Science.

[46] Ohoka N., Yoshii S., Hattori T., Onozaki K., Hayashi H. (2005). *TRB3* a novel ER stress-inducible gene, is induced *via* ATF4–CHOP pathway and is involved in cell death. EMBO J.

[47] Jacques S., van der Sloot A.M., Huard C.C. (2020). Imipridone anticancer compounds ectopically activate the ClpP protease and represent a new scaffold for antibiotic development. Genetics.

[48] Baker T.A., Sauer R.T. (2012). ClpXP, an ATP-powered unfolding and protein-degradation machine. Biochim Biophys Acta.

[49] Fischer F., Langer J.D., Osiewacz H.D. (2015). Identification of potential mitochondrial CLPXP protease interactors and substrates suggests its central role in energy metabolism. Sci Rep.

[50] Szczepanowska K., Maiti P., Kukat A. (2016). CLPP coordinates mitoribosomal assembly through the regulation of ERAL1 levels. EMBO J.

[51] Bhandari V., Wong K.S., Zhou J.L., Mabanglo M.F., Batey R.A., Houry W.A. (2018). The role of ClpP protease in bacterial pathogenesis and human diseases. ACS Chem Biol.

[52] Bonner E.R., Waszak S.M., Grotzer M.A., Mueller S., Nazarian J. (2021). Mechanisms of imipridones in targeting mitochondrial metabolism in cancer cells. Neuro Oncol.

[53] Greer Y.E., Hernandez L., Fennell E.M.J. (2022). Mitochondrial matrix protease ClpP agonists inhibit cancer stem cell function in breast cancer cells by disrupting mitochondrial homeostasis. Cancer Res Commun.

[54] Jackson E.R., Persson M.L., Fish C.J. (2024). A review of current therapeutics targeting the mitochondrial protease ClpP in diffuse midline glioma, H3 K27-altered. Neuro Oncol.

[55] Przystal J.M., Cianciolo Cosentino C., Yadavilli S. (2022). Imipridones affect tumor bioenergetics and promote cell lineage differentiation in diffuse midline gliomas. Neuro Oncol.

[56] Pruss M., Dwucet A., Tanriover M. (2020). Dual metabolic reprogramming by ONC201/TIC10 and 2-Deoxyglucose induces energy depletion and synergistic anti-cancer activity in glioblastoma. Br J Cancer.

[57] Wu J.C., Huang C.C., Wang P.W. (2023). ONC201 suppresses neuroblastoma growth by interrupting mitochondrial function and reactivating nuclear ATRX expression while decreasing MYCN. Int J Mol Sci.

[58] Dorsam R.T., Gutkind J.S. (2007). G-protein-coupled receptors and cancer. Nat Rev Cancer.

[59] Lappano R., Maggiolini M. (2011). G protein-coupled receptors: novel targets for drug discovery in cancer. Nat Rev Drug Discov.

[60] Conflitti P., Lyman E., Sansom M.S.P. (2025). Functional dynamics of G protein-coupled receptors reveal new routes for drug discovery. Nat Rev Drug Discov.

[61] Li J., Zhu S., Kozono D. (2014). Genome-wide shRNA screen revealed integrated mitogenic signaling between dopamine receptor D2 (DRD2) and epidermal growth factor receptor (EGFR) in glioblastoma. Oncotarget.

[62] Sachlos E., Risueño R.M., Laronde S. (2012). Identification of drugs including a dopamine receptor antagonist that selectively target cancer stem cells. Cell.

[63] Caragher S.P., Shireman J.M., Huang M. (2019). Activation of dopamine receptor 2 prompts transcriptomic and metabolic plasticity in glioblastoma. J Neurosci.

[64] Cherubini E., Di Napoli A., Noto A. (2016). Genetic and functional analysis of polymorphisms in the human dopamine receptor and transporter genes in small cell lung cancer. J Cell Physiol.

[65] Bartek J., Hodny Z. (2014). Dopamine signaling: target in glioblastoma. Oncotarget.

[66] Free R.B., Cuoco C.A., Xie B. (2021). Pharmacological characterization of the imipridone anticancer drug ONC201 reveals a negative allosteric mechanism of action at the D2 dopamine receptor. Mol Pharmacol.

[67] Beaulieu J.M., Espinoza S., Gainetdinov R.R. (2015). Dopamine receptors–IUPHAR review 13. Br J Pharmacol.

[68] Beom S., Cheong D., Torres G., Caron M.G., Kim K.M. (2004). Comparative studies of molecular mechanisms of dopamine D2 and D3 receptors for the activation of extracellular signal-regulated kinase. J Biol Chem.

[69] Beaulieu J.M., Tirotta E., Sotnikova T.D. (2007). Regulation of Akt signaling by D_2_ and D_3_Dopamine ReceptorsIn vivo. J Neurosci.

[70] Caragher S.P., Hall R.R., Ahsan R., Ahmed A.U. (2018). Monoamines in glioblastoma: complex biology with therapeutic potential. Neuro Oncol.

[71] He Y., Li J., Koga T. (2021). Epidermal growth factor receptor as a molecular determinant of glioblastoma response to dopamine receptor D2 inhibitors. Neuro Oncol.

[72] He L., Bhat K., Ioannidis A. (2021). Effects of the DRD2/3 antagonist ONC201 and radiation in glioblastoma. Radiother Oncol.

[73] Zhang Y., Zhou L., Safran H. (2021). EZH2i EPZ-6438 and HDACi vorinostat synergize with ONC201/TIC10 to activate integrated stress response, DR5, reduce H3K27 methylation, ClpX and promote apoptosis of multiple tumor types including DIPG. Neoplasia.

[74] Xin D.E., Liao Y., Rao R. (2024). Chaetocin-mediated SUV39H1 inhibition targets stemness and oncogenic networks of diffuse midline gliomas and synergizes with ONC201. Neuro Oncol.

[75] Miciaccia M., Rizzo F., Centonze A. (2024). Harmaline to human mitochondrial caseinolytic serine protease activation for pediatric diffuse intrinsic pontine glioma treatment. Pharmaceuticals.

[76] de la Nava D., Ausejo-Mauleon I., Laspidea V. (2024). The oncolytic adenovirus Delta-24-RGD in combination with ONC201 induces a potent antitumor response in pediatric high-grade and diffuse midline glioma models. Neuro Oncol.

[77] Zhou L., Zhang L., Zhang J. (2025). Imipridones ONC201/ONC206 + RT/TMZ triple (IRT) therapy reduces intracranial tumor burden, prolongs survival in orthotopic IDH-WT GBM mouse model, and suppresses MGMT. Oncotarget.

[78] Liguori N.R., Sanchez Sevilla Uruchurtu A., Zhang L. (2022). Preclinical studies with ONC201/TIC10 and lurbinectedin as a novel combination therapy in small cell lung cancer (SCLC). Am J Cancer Res.

[79] Ding E. (2025). Small cell lung cancer and prostate cancer cells with varying neuroendocrine differentiation markers show sensitivity to imipridone ONC201/TIC10. Am J Transl Res.

[80] Rumman M., Buck S., Polin L., Dzinic S., Boerner J., Winer I.S. (2021). ONC201 induces the unfolded protein response (UPR) in high- and low-grade ovarian carcinoma cell lines and leads to cell death regardless of platinum sensitivity. Cancer Med.

[81] Kou X., Ding H., Li L., Chao H. (2021). Caseinolytic protease P (CLPP) activated by ONC201 inhibits proliferation and promotes apoptosis in human epithelial ovarian cancer cells by inducing mitochondrial dysfunction. Ann Transl Med.

[82] Fan Y., Wang J., Fang Z. (2022). Anti-tumor and anti-invasive effects of ONC201 on ovarian cancer cells and a transgenic mouse model of serous ovarian cancer. Front Oncol.

[83] Di Cristofano F.R., Fong M.W., Huntington K.E., Carneiro B.A., Zhou L., El-Deiry W.S. (2023). Synergistic activity of ABT-263 and ONC201/TIC10 against solid tumor cell lines is associated with suppression of anti-apoptotic Mcl-1, BAG3, pAkt, and upregulation of pro-apoptotic Noxa and Bax cleavage during apoptosis. Am J Cancer Res.

[84] Ralff M.D., Kline C.L.B., Küçükkase O.C. (2017). ONC201 demonstrates antitumor effects in both triple-negative and non–triple-negative breast cancers through TRAIL-dependent and TRAIL-independent mechanisms. Mol Cancer Therapeut.

[85] Ralff M.D., Jhaveri A., Ray J.E. (2020). TRAIL receptor agonists convert the response of breast cancer cells to ONC201 from anti-proliferative to apoptotic. Oncotarget.

[86] Lim B., Peterson C.B., Davis A. (2021). ONC201 and an MEK inhibitor trametinib synergistically inhibit the growth of triple-negative breast cancer cells. Biomedicines.

[87] Fennell E.M.J., Aponte-Collazo L.J., Pathmasiri W. (2023). Multi-omics analyses reveal ClpP activators disrupt essential mitochondrial pathways in triple-negative breast cancer. Front Pharmacol.

[88] Farmaki E., Nath A., Emond R. (2023). ONC201/TIC10 enhances durability of mTOR inhibitor everolimus in metastatic ER+ breast cancer. Elife.

[89] Amoroso F., Glass K., Singh R. (2021). Modulating the unfolded protein response with ONC201 to impact on radiation response in prostate cancer cells. Sci Rep.

[90] Wu L.J. (2024). Synergistic combination therapy with ONC201 or ONC206, and enzalutamide or darolutamide in preclinical studies of castration-resistant prostate cancer. Am J Cancer Res.

[91] Zhang Y. (2025). Reduced EZH1/2 expression in imipridone-treated cells correlates with synergy following combinations with EZH1/2 or HDAC inhibitors in diffuse glioma and other tumors. Am J Cancer Res.

[92] Jin Z.Z., Wang W., Fang D.L., Jin Y.J. (2016). mTOR inhibition sensitizes ONC201-induced anti-colorectal cancer cell activity. Biochem Biophys Res Commun.

[93] Wagner J., Kline C.L., Zhou L., Khazak V., El-Deiry W.S. (2024). Correction: Anti-tumor effects of ONC201 in combination with VEGF-inhibitors significantly impacts colorectal cancer growth and survival *in vivo* through complementary non-overlapping mechanisms. J Exp Clin Cancer Res.

[94] Madka V., De La Cruz A., Pathuri G. (2022). Oral administration of TRAIL-inducing small molecule ONC201/TIC10 prevents intestinal polyposis in the *Apc*^min/+^ mouse model. Am J Cancer Res.

[95] Fang Z., Wang J., Clark L.H. (2018). ONC201 demonstrates anti-tumorigenic and anti-metastatic activity in uterine serous carcinoma in vitro. Am J Cancer Res.

[96] Pierce S.R., Fang Z., Yin Y. (2021). Targeting dopamine receptor D2 as a novel therapeutic strategy in endometrial cancer. J Exp Clin Cancer Res.

[97] Tu Y.S., He J., Liu H. (2017). The imipridone ONC201 induces apoptosis and overcomes chemotherapy resistance by up-regulation of BIM in multiple myeloma. Neoplasia.

[98] Zhang Q., Wang H., Ran L., Zhang Z., Jiang R. (2016). The preclinical evaluation of TIC10/ONC201 as an anti-pancreatic cancer agent. Biochem Biophys Res Commun.

[99] Xu J., Zhou J.Y., Wei W.Z., Wu G.S. (2010). Activation of the Akt survival pathway contributes to TRAIL resistance in cancer cells. PLoS One.

[100] Pimentel J.M., Zhou J.Y., Wu G.S. (2023). Regulation of programmed death ligand 1 (PD-L1) expression by TNF-related apoptosis-inducing ligand (TRAIL) in triple-negative breast cancer cells. Mol Carcinog.

[101] Kumar V., Sethi B., Staller D.W., Shrestha P., Mahato R.I. (2024). Gemcitabine elaidate and ONC201 combination therapy for inhibiting pancreatic cancer in a KRAS mutated syngeneic mouse model. Cell Death Discov.

[102] Pimentel J.M., Zhou J.Y., Kim S., Gurdziel K., Wu G.S. (2023). The role of the immune response and inflammatory pathways in TNF-related apoptosis-inducing ligand (TRAIL) resistance in triple-negative breast cancer cells. Am J Cancer Res.

[103] Jackson E.R., Duchatel R.J., Staudt D.E. (2023). ONC201 in combination with paxalisib for the treatment of H3K27-altered diffuse midline glioma. Cancer Res.

[104] Wagner J., Kline C.L., Zhou L. (2018). Dose intensification of TRAIL-inducing ONC201 inhibits metastasis and promotes intratumoral NK cell recruitment. J Clin Invest.

[105] Stein M.N., Malhotra J., Tarapore R.S. (2019). Safety and enhanced immunostimulatory activity of the DRD2 antagonist ONC201 in advanced solid tumor patients with weekly oral administration. J Immunother Cancer.

[106] Huntington K.E., Louie A., Zhou L., El-Deiry W.S. (2021). A high-throughput customized cytokinome screen of colon cancer cell responses to small-molecule oncology drugs. Oncotarget.

[107] Ghandali M., Huntington K.E., Srinivasan P. (2023). Abstract 1066: PARP inhibitor rucaparib in combination with imipridones ONC201 or ONC212 demonstrates preclinical synergy against BRCA1/2-deficient breast, ovarian, and prostate cancer cells. Cancer Res.

[108] Chuang H.C., Tsai M.H., Chuang J.H., Hong Y.T., Chien C.Y., Chou M.H. (2025). ONC201 enhances the cytotoxic effect of cisplatin through ATF3/ATF4/CHOP in head and neck squamous cell carcinoma cells. Oncogenesis.

[109] Lee Y.S., Zhou L., Azzoli C., El-Deiry W.S. (2022). Abstract 4056: ONC201 in combination with carboplatin and etoposide as a novel triple drug treatment for small cell lung cancer (SCLC). Cancer Res.

[110] Stein M.N., Bertino J.R., Kaufman H.L. (2017). First-in-human clinical trial of oral ONC201 in patients with refractory solid tumors. Clin Cancer Res.

[111] Cantor E., Wierzbicki K., Tarapore R.S. (2022). Serial H3K27M cell-free tumor DNA (cf-tDNA) tracking predicts ONC201 treatment response and progression in diffuse midline glioma. Neuro Oncol.

[112] Ekhator C., Rak R., Tadipatri R., Fonkem E., Grewal J. (2022). A single-center experience of dopamine antagonist ONC201 for recurrent histone H3 Lysine 27-to-Methionine (H3K27M)-Mutant glioblastoma in adults. Cureus.

[113] Gardner S.L., Tarapore R.S., Allen J. (2022). Phase I dose escalation and expansion trial of single agent ONC201 in pediatric diffuse midline gliomas following radiotherapy. Neurooncol Adv.

[114] Odia Y., Koschmann C., Vitanza N.A. (2024). Safety and pharmacokinetics of ONC201 (dordaviprone) administered two consecutive days per week in pediatric patients with H3 K27M-mutant glioma. Neuro Oncol.

[115] Arrillaga-Romany I., Chi A.S., Allen J.E., Oster W., Wen P.Y., Batchelor T.T. (2017). A phase 2 study of the first imipridone ONC201, a selective DRD2 antagonist for oncology, administered every three weeks in recurrent glioblastoma. Oncotarget.

[116] Chi A.S., Stafford J.M., Sen N. (2017). Exth-42. h3 k27m mutant gliomas are selectively killed by onc201, a small molecule inhibitor of dopamine receptor d2. Neuro Oncol.

[117] Chi A.S., Tarapore R.S., Hall M.D. (2019). Pediatric and adult H3 K27M-mutant diffuse midline glioma treated with the selective DRD2 antagonist ONC201. J Neuro Oncol.

[118] Hall M.D., Odia Y., Allen J.E. (2019). First clinical experience with DRD2/3 antagonist ONC201 in H3 K27M–mutant pediatric diffuse intrinsic pontine glioma: a case report. J Neurosurg Pediatr.

[119] Arrillaga-Romany I., Odia Y., Prabhu V.V. (2020). Biological activity of weekly ONC201 in adult recurrent glioblastoma patients. Neuro Oncol.

[120] Tanrıkulu B., Yaşar A.H., Canpolat C. (2023). Preliminary findings of german-sourced ONC201 treatment in H3K27 altered pediatric pontine diffuse midline gliomas. J Neuro Oncol.

[121] Anderson P.M., Trucco M.M., Tarapore R.S. (2022). Phase II study of ONC201 in neuroendocrine tumors including pheochromocytoma-paraganglioma and desmoplastic small round cell tumor. Clin Cancer Res.

[122] Atkins S.L.P., Greer Y.E., Jenkins S. (2023). A single-arm, open-label phase II study of ONC201 in recurrent/refractory metastatic breast cancer and advanced endometrial carcinoma. Oncologist.

[123] Almhanna K., Breakstone R., Raufi A. (2024). Nivolumab plus ONC201 plus in microsatellite stable (MSS) metastatic colorectal cancer (mCRC) patients: a brown university oncology research group phase Ib/II study (BrUOG379). Transl Gastroenterol Hepatol.

[124] Arrillaga-Romany I., Gardner S.L., Odia Y. (2024). ONC201 (dordaviprone) in recurrent H3 K27M–mutant diffuse midline glioma. J Clin Oncol.

[125] Arrillaga-Romany I., Lassman A., McGovern S.L. (2024). ACTION: a randomized phase 3 study of ONC201 (dordaviprone) in patients with newly diagnosed H3 K27M-mutant diffuse glioma. Neuro Oncol.

[126] Venneti S., Kawakibi A.R., Ji S. (2023). Clinical efficacy of ONC201 in H3K27M-Mutant diffuse midline gliomas is driven by disruption of integrated metabolic and epigenetic pathways. Cancer Discov.

[127] Di Carlo D., Annereau M., Vignes M. (2025). Real life data of ONC201 (dordaviprone) in pediatric and adult H3K27-altered recurrent diffuse midline glioma: results of an international academia-driven compassionate use program. Eur J Cancer.

[128] Borsuk R., Zhou L., Chang W.I. (2021). Potent preclinical sensitivity to imipridone-based combination therapies in oncohistone H3K27M-mutant diffuse intrinsic pontine glioma is associated with induction of the integrated stress response, TRAIL death receptor DR5, reduced ClpX and apoptosis. Am J Cancer Res.

[129] Zhang Y., Huang Y., Yin Y. (2020). ONC206, an imipridone derivative, induces cell death through activation of the integrated stress response in serous endometrial cancer *in vitro*. Front Oncol.

[130] Tucker K., Yin Y., Staley S.A. (2022). ONC206 has anti-tumorigenic effects in human ovarian cancer cells and in a transgenic mouse model of high-grade serous ovarian cancer. Am J Cancer Res.

[131] Nguyen T.T.T., Shang E., Schiffgens S. (2022). Induction of synthetic lethality by activation of mitochondrial ClpP and inhibition of HDAC1/2 in glioblastoma. Clin Cancer Res.

[132] Lev A., Lulla A.R., Wagner J. (2017). Anti-pancreatic cancer activity of ONC212 involves the unfolded protein response (UPR) and is reduced by IGF1-R and GRP78/BIP. Oncotarget.

[133] Chattopadhyay C., Roszik J., Bhattacharya R. (2024). Imipridones inhibit tumor growth and improve survival in an orthotopic liver metastasis mouse model of human uveal melanoma. Br J Cancer.

[134] Ferrarini I., Louie A., Zhou L., El-Deiry W.S. (2021). ONC212 is a novel mitocan acting synergistically with glycolysis inhibition in pancreatic cancer. Mol Cancer Therapeut.

[135] Nii T., Prabhu V.V., Ruvolo V. (2019). Imipridone ONC212 activates orphan G protein-coupled receptor GPR132 and integrated stress response in acute myeloid leukemia. Leukemia.

[136] Su Y., Carter J.L., Li X. (2024). The imipridone ONC213 targets α-Ketoglutarate dehydrogenase to induce mitochondrial stress and suppress oxidative phosphorylation in acute myeloid leukemia. Cancer Res.

[137] Carter J.L., Su Y., Al-Antary E.T. (2025). ONC213: a novel strategy to resensitize resistant AML cells to venetoclax through induction of mitochondrial stress. J Exp Clin Cancer Res.

[138] Szász Z., Takács A., Kalabay M. (2025). Comparative study of the anti-tumour effects of the imipridone, ONC201 and its fluorinated analogues on pancreatic cancer cell line. Sci Rep.

[139] Giarrizzo M., LaComb J.F., Patel H.R. (2024). TR-107, an agonist of caseinolytic peptidase proteolytic subunit, disrupts mitochondrial metabolism and inhibits the growth of human colorectal cancer cells. Mol Cancer Therapeut.

[140] Jackson E.R., Duchatel R.J., Mannan A. (2020). Akt signaling drives resistance to onc201 in diffuse intrinsic pontine glioma (Dipg). Neuro Oncol.

[141] Dwucet A., Pruss M., Cao Q. (2021). ONC201/TIC10 is empowered by 2-deoxyglucose and causes metabolic reprogramming in medulloblastoma cells *in vitro* independent of C-myc expression. Front Cell Dev Biol.

[142] Ji S., Mota M., Natarajan S.K. (2025). DMG-63. EGFR confers resistance to ONC201 in H3K27M-mutant diffuse midline glioma. Neuro Oncol Pediatr.

[143] Savary C., Persson M., Jackson E.R. (2025). Single-cell analysis of ONC201 and paxalisib resistance mechanisms in diffuse intrinsic pontine glioma. Neuro Oncol Pediatr.

[144] Maranto C., Foster S., Lee A., Sethna P., Allen J.E., Prabhu V.V. (2024). Abstract A014: ClpP drives efficacy and mediates acquired resistance with imipridones ONC201 and ONC206 in glioma *in vitro*. Cancer Res.

[145] Shrestha P., Ghanwatkar Y., Mahto S., Pramanik N., Mahato R.I. (2024). Gemcitabine–lipid conjugate and ONC201 combination therapy effectively treats orthotopic pancreatic tumor-bearing mice. ACS Appl Mater Interfaces.

[146] Fatima N., Shen Y., Crassini K. (2024). The CIpP activator, TR-57, is highly effective as a single agent and in combination with venetoclax against CLL cells *in vitro*. Leuk Lymphoma.

[147] Bhatt V.R., Wichman C., Bouska A.C. (2022). Novel first-in-class drug ONC201 as a post-transplant maintenance for AML and MDS: a phase I trial in progress. Blood.

[148] Weissenrieder J.S., Reed J.L., Moldovan G.L. (2021). Antipsychotic drugs elicit cytotoxicity in glioblastoma multiforme in a calcium-dependent, non-D_2_ receptor-dependent, manner. Pharmacol Res Perspect.

[149] Batistella G., Costa F.D., Freddi T., Wojtynek N., Korones D. (2025). CTP-15. Dordaviprone (ONC201) in a pediatric patient with recurrent H3 K27M-mutant diffuse glioma and leptomeningeal disease. Neuro Oncol.

[150] Kawakibi A.R., Tarapore R., Gardner S. (2022). Clinical efficacy and predictive biomarkers of onc201 in h3k27m-mutant diffuse midline glioma. Neuro Oncol.

